# Interventional management of chest wall sarcomatoid carcinoma mimicking breast cancer metastasis: A case report

**DOI:** 10.1097/MD.0000000000044090

**Published:** 2025-08-29

**Authors:** Qiyu Zhang, Qiongyu Liang, Jiapeng Sun, Chi Xu

**Affiliations:** a Department of Interventional Treatment, Beijing No. 6 Hospital, Beijing, China; b Department of Radiology, Peking Union Medical College Hospital, Chinese Academy of Medical Sciences, Beijing, China.

**Keywords:** breast cancer, case reports, interventional therapy, multiple/second primary malignancy

## Abstract

**Rationale:**

Breast cancer patients have an increased risk of developing second primary malignancies, with lung cancer accounting for approximately 5% of cases. Differentiating between metastatic disease and a second primary malignancy remains a diagnostic challenge.

**Patient concerns:**

A 45-year-old woman with a history of triple-negative breast cancer presented with a newly detected chest wall nodule during routine follow-up.

**Diagnoses:**

Contrast-enhanced imaging suggested metastatic disease. However, biopsy and immunohistochemistry confirmed a second primary lesion, consistent with either pulmonary sarcomatoid carcinoma or chest wall sarcoma.

**Interventions:**

The patient underwent a multimodal interventional regimen, including transarterial embolization followed immediately by radiofrequency ablation.

**Outcomes:**

At 1 month post-treatment, follow-up imaging demonstrated significant tumor shrinkage with no evidence of local recurrence.

**Lessons:**

This case underscores the importance of comprehensive diagnostic evaluation to distinguish metastatic disease from second primary malignancies in breast cancer patients. It also highlights the potential of interventional therapy as a minimally invasive treatment option for inoperable tumors, with multidisciplinary management being essential to optimize outcomes and prognosis.

## 1. Introduction

Breast carcinoma is one of the most common malignant tumors worldwide.^[1]^ With advancements in treatment and early detection, the survival rates of patients with breast cancer have steadily increased. Approximately 10% of breast cancer patients who survive for more than 10 years develop a second primary cancer, with lung cancer accounting for about 5% of cases. Double primary cancers are defined as the simultaneous occurrence of 2 distinct malignant tumors in different anatomical locations, each with a unique histological origin. These tumors fall under the broader category of multiple primary cancers, which involves cases where separate malignancies arise independently rather than as metastases from a single primary tumor. Accurately distinguishing metastatic lesions from second primary cancers and formulating individualized treatment strategies has become a significant challenge in clinical practice.

We presented a case of a 45-year-old woman diagnosed with a triple-negative breast cancer (TNBC) who developed a thoracic lesion initially suspected to be a metastasis. Diagnosis of primary pulmonary sarcomatoid carcinoma or chest wall sarcoma was established through histopathological and immunohistochemical analyses. The patient achieved favorable local control through a novel combination of transarterial embolization (TAE) and thermal ablation, highlighting the crucial role of interventional techniques in managing complex dual malignancies.

## 2. Case report/case presentation

**Figure 1.**
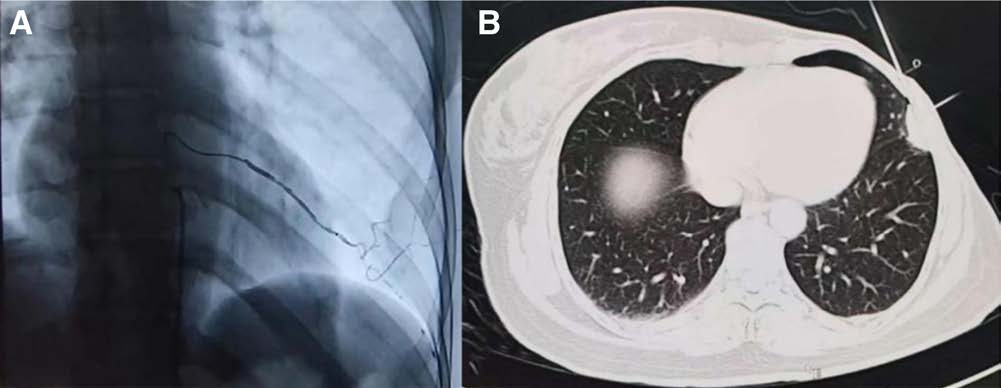
Intraoperative imaging of combined transarterial embolization and radiofrequency ablation. (A) Fluoroscopic image demonstrating selective catheterization of the tumor-feeding intercostal artery and embolization with lipiodol and microspheres during transarterial embolization. (B) Axial CT image obtained during percutaneous radiofrequency ablation, showing electrode placement within the lesion and the surrounding hydro-dissection fluid to protect adjacent pleura and intercostal nerves.

**Figure 2.**
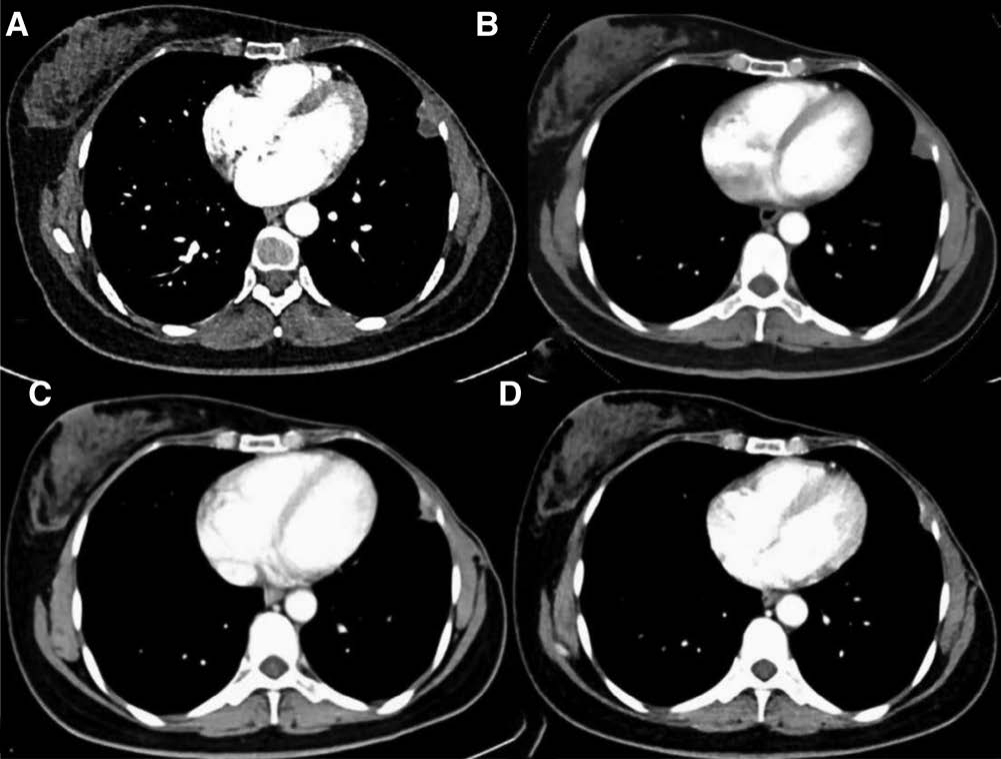
Serial contrast-enhanced axial CT images of the chest wall lesion. (A) Baseline (pretreatment), (B) 1-month posttreatment, (C) 3 months posttreatment, (D) 6 months posttreatment.

Written informed consent was obtained from the patient before the treatment and the preparation of this case report. This case report followed the CARE guideline. We report the case of a 45-year-old female with a family history of breast cancer who was admitted to the hospital after discovering a lump in the outer quadrant of her left breast, leading to a punch biopsy. Pathological analysis confirmed the diagnosis of invasive breast carcinoma, which was negative for estrogen receptor (ER), progesterone receptor (PR), and human epidermal growth factor receptor 2 (HER2). Based on this diagnosis, she received neoadjuvant chemotherapy consisting of docetaxel (75 mg/m²), doxorubicin (50 mg/m²), cyclophosphamide (500 mg/m²), every 3 weeks for 6 cycles, followed by a modified radical mastectomy in November 2018. Postsurgical pathology suggested no definite residual tumor in the breast tissue, with focal interstitial fibrous tissue hyperplasia and foam cell aggregates. After the modified radical mastectomy, the patient underwent 25 cycles of radiotherapy. In April 2024, during a routine follow-up for breast cancer, a chest computerized tomography (CT) scan detected a nodule on the left chest wall measuring approximately 13 × 8 mm in size. Continued observation was recommended. In August 2024, follow-up imaging revealed an increase in nodule size to approximately 23 × 14 mm (shown in Figure 2A). Contrast-enhanced imaging demonstrated peripheral enhancement and invasion of the adjacent rib, raising a strong suspicion of malignancy, with a high likelihood of breast cancer metastasis. To clarify the diagnosis and guide further clinical treatment, the patient underwent multimodal interventional treatment at the Department of Interventional Treatment in September 2024. The procedure was performed as follows: First, under digital subtraction angiography guidance, a microcatheter was selectively advanced into the tumor-supplying intercostal artery. Lipiodol and embolic microspheres (Embosphere, 300–500 µm) were injected to mark and embolize the lesion (shown in Figure 1A). Following embolization, a tissue specimen was obtained via CT-guided percutaneous biopsy. Subsequently, radio frequency ablation (RFA)was performed to target the lesion (shown in Figure 1B). The patient received adequate local anesthesia prior to the RFA procedure. A 2-electrode upper and lower cross-needle strategy was employed, with an ablation power set between 40 and 60 W. The ablation lasted for approximately 4 minutes. During the thermal ablation process, we manually injected subpleural isolation fluid to create a protective barrier, physically separating the tumor from the mural pleura and intercostal nerves. This approach helped minimize thermal damage to sensitive structures and reduce procedural pain. Examination revealed sarcomatoid carcinoma or undifferentiated sarcoma. Immunohistochemistry showed the lesion to be PR- and HER2-negative, with weak ER positivity and a low Ki-67 proliferation index of 10%. It also expressed TRPS1 but was negative for GATA3, AE1/AE3, and CAM5.2. Based on the combination of immunohistochemistry findings and imaging features, the final diagnosis was a bilateral primary malignant tumor. One month after treatment, a contrast-enhanced CT scan indicated a significant reduction in the size of the lesion, which measured approximately 19 × 9 mm (shown in Figure 2B). Although the chest lesion was well-controlled, its clinical features were more suggestive of pleural metastasis from breast cancer. To determine the appropriate systemic treatment strategy for breast cancer, a reevaluation of the pathology was requested. In December 2024, a pathological biopsy of the chest nodule was performed, demonstrating fibrous tissue with carbon deposition and associated flaky necrosis. Following clinical evaluation, the patient was administered the albumin-bound paclitaxel plus cisplatin chemotherapy regimen. By February 2025, a reexamination of the nodule showed a significant reduction in size to approximately 13 × 7 mm (shown in Figure 2C), much smaller than prior treatment, with no evidence of peripheral or distant metastatic foci. This case demonstrates effective local control of the lesion through interventional treatment, offering new insights into the comprehensive diagnosis and management of multiple primary malignancies.

## 3. Discussion

Advancements in breast cancer treatment have significantly prolonged patient survival and increased the detection rates of second primary cancers.^[2]^ Risk factors associated with these cancers include lifestyle and environmental factors, such as smoking and alcohol consumption, tumor treatments, including chemotherapy and radiotherapy, and genetic factors, such as BRCA1/2 mutations. Common concurrent cancers include endometrial, lung, and colorectal cancers. Lung cancer is particularly notable due to its dual role as both a prevalent second primary cancer and a common metastatic site for breast cancer. The substantial overlap in imaging and clinical manifestations between second primary lung cancer and metastatic lung cancer often leads to misdiagnosis and underdiagnosis, which can delay timely and precise treatment. Radiation therapy, a well-recognized risk factor for second primary lung cancer in breast cancer patients, contributes to damage to normal lung tissue and has potential oncogenic effects.^[3]^ These findings highlight the importance of clinicians closely monitoring risk factors and conducting regular follow-up assessments for breast cancer patients throughout their treatment to identify potential second primary cancers at an early stage. In this case report, the female patient underwent multiple radiotherapy treatments for breast cancer. Pathological examination revealed that both breast cancer and the chest lesions exhibited independent pathological features, meeting the diagnostic criteria for multiple/second primary malignancy. This not only provides a solid pathological foundation for diagnosing multiple primary cancers but also emphasizes the critical need to differentiate between primary and metastatic tumors in clinical practice.

Pathological confirmation remains the gold standard for diagnosing breast cancer metastasis. Pathological evaluation not only determines the benign or malignant nature of the tumor but also identifies the tissue origin of the new malignant lesion. Furthermore, it allows for reassessment of ER, PR, and HER2 status, which is crucial for optimizing treatment strategies. As a minimally invasive technique, image-guided puncture biopsy offers high precision and low invasiveness. When combined with real-time CT, it enhances sampling accuracy, particularly for inoperable patients, providing a safe diagnostic approach.^[4]^ The biopsy samples not only confirm the diagnosis but also serve as a strong foundation for molecular profiling and the development of targeted or immunotherapeutic treatment strategies.^[5]^

The clinical team from the thoracic surgery department recommended performing surgical resection for this patient’s new chest lesion, considering the patient’s overall condition and local tumor control. Therefore, we decided to adopt a combined treatment approach of TAE combined with RFA, thus avoiding unnecessary risks. Although there remains a diagnostic controversy regarding whether the primary tumor originated from the chest wall or lung tissue, the combined treatment has demonstrated significant efficacy in lesion control. Postoperative follow-up data shows a >50% reduction in lesion volume at 2 months posttreatment, with no recurrence observed on imaging at 10 months posttreatment.^[6]^ Theoretically, TAE can effectively block the tumor’s blood supply, therefore reducing the risk of bleeding during the ablation process. Additionally, the iodized oil facilitates the detection of small lesions, also tracing under CT to localize lesions.^[7]^ Although thermal ablation has been associated with higher complication rates compared to cryoablation, Xu et al reported that its complication rate was approximately 4 times higher, primarily due to pain and discomfort.^[8]^ Hydro-dissection is an effective technique to relieve the pain and decrease the occurrence of pneumothorax.^[9]^ In this case report, we employed the hydro-dissection technique by injecting isolation fluid to facilitate the RFA procedure, significantly reducing pain during treatment. Furthermore, during the thermal ablation process, the heat generated is carried away by blood flow, potentially reducing treatment effectiveness. However, employing embolization before the RFA procedure blocks blood flow, minimizing heat loss and significantly enhancing the concentration of thermal energy, thus minimizing the effective ablation area and improving treatment efficiency.^[10]^

Currently, the combined treatment has gained significant clinical evidence in the treatment of liver and kidney tumors/conditions. However, clinical studies focusing on thoracic tumors remain limited. A clinical study by the Ven Fong Z team on 32 patients with colorectal cancer liver metastasis demonstrated that the TAE-RFA approach not only exhibited high safety, with a major complication rate of <5%, but also improved the 5-year survival rate of patients to 10%.^[11]^ Notably, a study by LaRussa S and colleagues innovatively utilized a single combination treatment to successfully address 11 patients with renal cell carcinoma tumors exceeding 3 cm in diameter. The average postoperative follow-up period was 13 months, with no local recurrence, thus providing high-level evidence for the efficacy of combined interventional treatments in advanced tumors.^[12]^ This innovation highlights the potential benefits of this combined approach in mitigating local recurrence and distant metastasis, thereby offering valuable insights for local control and overall management of advanced tumors.^[13–15]^

For inoperable solitary chest lesions, interventional treatment presents dual benefits: it facilitates precise sampling for pathological confirmation while also enabling tumor reduction and local control.^[12,16]^ This strategy is especially advantageous for ambiguous diagnoses or highly malignant tumors, providing reliable diagnostic and therapeutic support for challenging cases.

Despite the strengths of this case report, several limitations need consideration. Despite the strengths of this case report, several limitations need consideration. First, without molecular profiling, we could not conclusively determine the tumor’s genetic or molecular mutations, which may have provided valuable insights into its biological behavior, potential precise therapeutic targets, or prognosis. The absence of data on key mutations, gene expression, or protein markers limited our ability to explore personalized treatment options and may have restricted the depth of diagnostic and prognostic interpretation. Second, since the patient received TACE combined ablation in September 2024, the longer term follow-up data are not currently available, hence limiting our ability to assess disease progression and overall effectiveness. Third, despite distinct pathological features, uncertainty persists regarding the chest lesion’s precise origin, whether it arose from the chest wall or represents pulmonary sarcomatoid carcinoma.

Recent transcriptomic and single-cell studies have identified IL27RA and TMEM71 as immune-related biomarkers predictive of therapeutic response in TNBC.^[17,18]^ Incorporating these markers into diagnostic algorithms could significantly improve discrimination between second primary malignancies and metastatic disease, which is a critical distinction underscored by the current case of a chest wall mass developing post-TNBC. Additionally, validated inflammation-nutrition indices (e.g., neutrophil–platelet/lymphocyte–hemoglobin ratio, modified Glasgow Prognostic Score) provide independent prognostic stratification in breast cancer.^[19,20]^ Integrating these multimodal biomarkers may refine therapeutic decisions regarding intensity of local interventions (such as embolization and radiofrequency ablation) versus systemic therapy. Synthesizing molecular diagnostics, prognostic indices, and interventional approaches within a multidisciplinary team framework, as demonstrated here, represents a promising strategy for personalized management of complex thoracic malignancies.

## 4. Patient perspective

With the patient’s consent, we asked this patient to provide a brief perspective reflecting her thoughts on the diagnosis and interventional treatment. She shared that receiving news about a potential new malignancy was initially overwhelming and anxious, especially given her history of breast cancer. However, she appreciated the clear communication and explanation provided by the multidisciplinary clinical team, which helped her understand the nature of the chest lesion. The minimally invasive approach, combined with the supportive and multidisciplinary care she received, made her feel safe and respected throughout the process. She expressed satisfaction with the outcome and gratitude for the care team’s efforts to minimize discomfort and ensure effective treatment.

## Author contributions

**Conceptualization:** Qiyu Zhang, Qiongyu Liang, Jiapeng Sun, Chi Xu.

**Data curation:** Qiyu Zhang.

**Investigation:** Qiyu Zhang, Qiongyu Liang, Jiapeng Sun.

**Methodology:** Qiongyu Liang.

**Project administration:** Qiongyu Liang, Chi Xu.

**Resources:** Qiyu Zhang, Jiapeng Sun, Chi Xu.

**Supervision:** Chi Xu.

**Validation:** Qiyu Zhang.

**Visualization:** Jiapeng Sun.

**Writing – original draft:** Qiyu Zhang, Qiongyu Liang, Jiapeng Sun.

**Writing – review & editing:** Qiyu Zhang, Qiongyu Liang, Jiapeng Sun, Chi Xu.
